# The effect of diet density on allometry in pullet growth and early egg production

**DOI:** 10.1016/j.psj.2023.103211

**Published:** 2023-10-18

**Authors:** Lieske van Eck, Adele Schouten, Syrena Powell, David Lamot, Henk Enting, Rene Kwakkel

**Affiliations:** ⁎ Cargill Animal Nutrition Innovation Center, NL-5334 LD Velddriel, the Netherlands; † Department of Animal Sciences, Wageningen University, NL-6700 AH Wageningen, the Netherlands

**Keywords:** pullet, allometry, growth, diet density

## Abstract

Understanding the effect of nutrition on pullet growth curves and body composition may help to design new feeding strategies that influence body composition and (long-term) laying performance. Therefore, this study examined the effect of nutrient density (low, medium or high metabolizable energy and essential amino acids), fed in the rearing phase until 17 wk of age, on Hy-Line white W80 pullet growth, body composition development and egg production performance until wk 35. Data were subjected to mixed model analyses. To determine a multiphasic allometric relationship between body components, an overall growth curve was established and inflection points were determined. The linear higher BW at the end of the rearing phase, due to increased diet density, was maintained during the peak production phase until wk 35. Egg production parameters were not affected by rearing diet density. Breast and body crude protein percentages were not influenced by dietary treatments, whereas body crude fat and abdominal fat pad percentages were linearly increased with diet density from early age onward. Body crude protein was initially deposited at the same rate as body dry matter. In a second phase of growth from wk 12 onward, crude protein deposition was lower than body dry matter deposition, but was not influenced by rearing diet. Body crude fat, on the other hand, initially grew at a lower rate than body dry matter, but increased in deposition rate during a second phase of growth starting at wk 2 to 5. Pullets fed the high density diet showed higher deposition of crude fat vs. dry matter as compared to pullets fed the medium density diets in the first phase until wk 2, but exhibited lower crude fat deposition in the second phase until wk 8. These results indicate that until approximately wk 12, crude protein deposition was mainly driving growth and was not influenced by diet density. From wk 5 to 6 onward, crude fat deposition relative to protein deposition increased and this was influenced by diet density from an early age.

## INTRODUCTION

In the last 50 yr, the lifetime egg production capacity of laying hens has increased tremendously from 220 eggs in 1960 to 500 eggs in 2019 (Martin, 1960; Anderson, 2019). This increase has been achieved through genetic selection, improved management, better feeding strategies and proper pullet development until 17 wk of age (Bain et al., 2016; Wang et al., 2017; Underwood et al., 2021). Nevertheless, how early development contributes to egg production is not yet fully understood. The majority of the studies into pullet development have focused on reaching a target body weight at the end of the rearing phase, which is positively correlated to higher egg mass production throughout lay (Bouvarel et al., 2011). Next to the absolute body weight of pullets, also their body composition (crude protein, crude ash, and crude fat) and thus body condition should be considered (Kwakkel et al., 1993). Crude protein is deposited into muscle, essential tissues and organs, whereas ash is used for skeletal structures and most of the crude fat is accumulated in adipose tissue.

Adipose tissue is important in maintaining body energy balance and can function as a reserve when nutrient intake is not sufficient to fulfill the energy needs of the hen (Barboza and Hume, 2006), for example during peak egg production. Therefore, a currently unknown minimum level of adipose tissue at the start of lay might be beneficial to support production. On the other hand, pullets with a high body fat condition at start of lay have been shown to have reduced subsequent laying rates (Milisits et al., 2015) and egg weights throughout the laying phase (Cheng et al., 1991). The percentage of pullet body fat might therefore have an optimum, after which egg production is negatively affected.

The body fat condition can be altered through nutrition and in general, a higher protein and energy intake increase pullet body weights and egg mass production (Summers and Leeson, 1994; Hussein et al., 1996; Oluwabiyi et al., 2022). The effect of nutrient intake on body composition growth was not measured in these experiments and has not been studied in modern pullets. In broiler breeder pullets, higher body weights also result in higher body fat percentages (van Emous et al., 2013). Moreover, a lower dietary crude protein to energy ratio during rearing caused a significant higher body fat percentage at 20 wk of age. Broiler breeder pullets are fed restrictedly to gain similar body weights, so these results are not directly applicable to ad libitum fed laying hen pullets, that also have a lower growth potential. It nevertheless indicates that lower crude protein intake with similar energy intake results in higher fat deposition. Also diet density influences body composition in broiler breeder pullets, with increased body fat percentages at the end of rearing when diets with a higher density (both energy and lysine, fed in the same ratio) are fed (de los Mozos et al., 2017).

Understanding the effect of nutrition on pullet growth curves and body composition may help to design new diet strategies to influence body composition and potentially improve (long-term) laying performance. Therefore, this study examined the effect of nutrient density (low, medium, or high metabolizable energy, essential amino acids and macro minerals) in rearing diets on egg-type pullet growth and body composition development. In comparison to pullets fed a lower nutrient density, we hypothesized that pullets fed higher nutrient density diets would have a higher absolute nutrient intake. As a consequence, these pullets would have an increased gain that resulted in a higher 17-wk body weight and increased fat deposition. During lay, pullets with the largest fat pad might have lower egg production but would not go into a negative energy balance, since the body fat content could buffer the normally observed negative energy balance due to inadequate intake level. To achieve the desired difference in growth and body composition, a common diet was used following the breed guidelines (Hy-Line, 2016) and multiplied by 0.9 and 1.1 to achieve the low and high density diets. It was expected that this difference would result in significant feed intake differences (thereby also changing the protein and energy intake). In the trial by Hussein et al. (1996), an energy difference of 10% fed in wk 15 to 18 resulted in significant feed intake differences. We expected that this difference would be amplified if the dietary treatments were fed throughout the rearing period. To determine if the effect was limited by a maximum or minimum dietary density level, the range was broadened and both increased and decreased around the medium density diet.

## MATERIALS AND METHODS

All procedures were approved by the Animal Welfare Committee of the Cargill Innovation Center (Velddriel) in accordance with Dutch laws and regulations on the execution of animal experiments (AVD220002016765).

### Animals and Housing

We studied the effect of a low, medium or high diet density on Hy-Line white W80 pullet growth. Dietary treatments with 3 nutrient densities (low, medium, and high) were fed to the pullets from 0 to 17 wk of age, after which all hens received a common diet until 35 wk of age. A total of 1,080 one-day-old pullets were obtained from a commercial hatchery (Kuikenbroederij Jip van den Broek, the Netherlands) and randomly divided over 24 floor pens with 45 pullets per pen at the Cargill Animal Nutrition Innovation Center (Velddriel, the Netherlands). Pens were divided over 8 blocks with 3 pens per block; treatments were randomly allocated within block. The research facility contained 4 rows of pens and blocks were arranged alongside the length of a row. During the first 17 wk of age, pullets were housed in pens of 2.58 m^2^ with a deep littered floor (flax) covering 33% of the pen and an elevated floor with plastic slats covering 66% of the pen. Pullets were vaccinated according to standard Dutch veterinary practices. At 17 wk of age, all hens were transferred to layer pens. Birds remained in the same experimental units and were moved keeping the same blocking structure, but now divided over 2 rooms with 12 pens in each room. Pens were 2.29 m^2^ and had a raised floor, consisting of plastic plates covered with a 2 cm layer of wood shavings and 270 cm length of nest box was available. Feed and water were provided ad libitum during the entire trial. Light and temperature schedules followed the Hy-Line breeder recommendations during the entire trial (Hy-Line, 2016).

### Diet Formulation

Breeder nutritional guidelines (Hy-Line, 2016) were used as reference for diet formulation. Nutrient composition of the low and high density diets were calculated by multiplying all minimum nutrient levels according to the management guide (Hy-Line, 2016) by 0.9 or 1.1 (Table 1). The medium density diet was created by mixing the high and low density diets to maintain a linear response in case of unintended production errors. Feed was provided in 6 phases in accordance with the breed recommendations: starter 1 (wk 0–3; crumble), starter 2 (wk 3–6; crumble), grower (wk 6–12; mash), developer (wk 12–15; mash), prelay (wk 15–17; mash) and peak (17–35; mash). Major ingredient shifts between treatments and phases were avoided by assuring a minimum similar inclusion level of raw materials of 75% between treatments. Before diet formulation, batches of corn, soybean meal, wheat middlings and oat hulls were reserved and analyzed in accordance with standard laboratory methods. The ingredients and diets were analyzed for: dry matter (**DM**; ISO 6496, 1999), crude protein (**CP**; ISO 16634-1, 2008), crude fat (**CF**; ISO 6492, 1999), ash (ISO 5984, 2002), calcium (ISO 27085, 2009), and phosphorus (ISO 27085, 2009). Diet formulation was based on digestibility and nutrient calculations provided by CVB, 2016, based on the analyzed nutrient levels of the raw materials. The diets were produced by Research Diet Services (the Netherlands).

**Table 1:** Ingredient and nutrient composition of the experimental diets.

Feeding phase		Starter 1, wk 0–3		Starter 2, wk 3–6		Grower, wk 6–12			Developer, wk 12–15			Prelay, wk 15–17			Lay		
Diet density		Low	Medium	High	Low	Medium	High	Low	Medium	High	Low	Medium	High	Low	Medium	High	All
**Ingredients composition, g/kg**																	

Corn		35.11	34.21	33.30	31.27	31.92	32.57	36.53	34.96	33.40	41.05	41.74	42.43	43.52	39.72	35.93	34.52
Wheat middlings		29.29	14.65	-	31.39	15.69	-	34.96	17.48	-	37.76	18.88	-	24.91	12.46	-	5.00
Soybean meal		23.58	28.38	33.17	20.36	24.92	29.48	17.65	21.68	25.70	11.02	16.15	21.29	16.68	20.67	24.66	24.77
Wheat		-	10.00	20.00	5.00	15.00	25.00	-	15.00	30.00	-	13.45	26.91	-	11.00	22.00	20.00
Oats husk meal		5.00	3.00	1.00	5.00	3.00	1.00	5.00	3.00	1.00	5.00	3.30	1.61	5.00	3.00	1.00	-
Soybean oil		2.61	4.72	6.84	2.63	4.45	6.27	1.68	2.99	4.30	1.00	1.67	2.35	1.69	3.90	6.12	2.51
Limestone, fine		1.55	1.73	1.91	1.58	1.76	1.94	1.62	1.81	1.99	1.63	1.82	2.00	-	-	-	-
Limestone, 50% coarse, 50% fine		-	-	-	-	-	-	-	-	-	-	-	-	5.63	5.63	6.27	-
Limestone, 60% coarse, 40% fine		-	-	-	-	-	-	-	-	-	-	-	-	-	-	-	10.17
Monocalciumphosphate		0.997	1.232	1.466	0.950	1.192	1.434	0.877	1.116	1.355	0.879	1.138	1.397	0.968	1.205	1.442	1.325
Sodiumbicarbonate		0.342	0.357	0.372	0.330	0.346	0.362	0.298	0.314	0.329	0.317	0.309	0.300	0.247	0.251	0.254	0.210
Salt		0.204	0.250	0.295	0.186	0.232	0.277	0.157	0.203	0.249	0.145	0.208	0.270	0.177	0.228	0.279	0.241
Potassium carbonate 52%		-	0.088	0.176	-	0.115	0.230	-	0.162	0.324	-	0.143	0.285	-	0.079	0.158	-
DL-Methionine		0.211	0.255	0.298	0.192	0.235	0.278	0.162	0.198	0.233	0.101	0.127	0.152	0.153	0.192	0.230	0.226
L-Lysine HCL		0.079	0.091	0.103	0.077	0.090	0.103	0.045	0.061	0.076	0.071	0.036	-	0.013	0.007	-	-
L-Threonine		0.022	0.035	0.048	0.016	0.029	0.041	0.005	0.019	0.032	0.010	0.005	-	-	0.007	0.013	0.023
L-Tryptophan		-	-	-	-	-	-	-	-	-	-	-	-	-	0.002	0.003	-
NSP^1^		0.010	0.010	0.010	0.010	0.010	0.010	0.010	0.010	0.010	0.010	0.010	0.010	0.010	0.010	0.010	0.010
Phytase^2^		0.005	0.005	0.005	0.005	0.005	0.005	0.005	0.005	0.005	0.005	0.005	0.005	0.005	0.005	0.005	0.005
Premix^3^		1.00	1.00	1.00	1.00	1.00	1.00	1.00	1.00	1.00	1.00	1.00	1.00	1.00	1.00	1.00	1.00

**Calculated chemical composition**																	

Dry matter	%	88.25	88.77	89.29	88.28	88.75	89.23	87.98	88.48	88.98	87.35	87.95	88.55	88.16	88.87	89.59	89.37
Crude protein	%	19.89	20.87	21.86	18.94	19.76	20.58	17.98	18.66	19.34	15.17	16.29	17.41	16.08	17.03	17.98	18.08
Crude fat	%	5.00	6.74	8.47	5.00	6.46	7.92	4.28	5.16	6.04	4.38	4.38	4.38	4.59	6.21	7.82	4.27
Ash	%	6.68	6.90	7.12	6.54	6.75	6.97	6.43	6.62	6.81	6.21	6.41	6.62	10.02	10.71	11.39	14.39
Calcium	%	0.90	1.00	1.10	0.90	1.00	1.10	0.90	1.00	1.10	0.90	1.00	1.10	2.43	2.70	2.97	4.20
Phosphorous	%	0.70	0.69	0.69	0.69	0.68	0.67	0.68	0.66	0.64	0.70	0.67	0.63	0.65	0.64	0.63	0.64
Available Phosphorous	%	0.45	0.50	0.55	0.44	0.49	0.54	0.42	0.47	0.52	0.42	0.47	0.52	0.43	0.48	0.53	0.51
Sodium	%	0.19	0.21	0.23	0.18	0.20	0.22	0.16	0.18	0.20	0.16	0.18	0.20	0.16	0.18	0.20	0.18
Potassium	%	1.08	1.07	1.06	1.04	1.03	1.01	1.02	1.01	1.00	0.92	0.91	0.90	0.89	0.88	0.87	0.84
Chloride	%	0.19	0.21	0.23	0.18	0.20	0.22	0.16	0.18	0.20	0.16	0.18	0.20	0.16	0.18	0.20	0.18
ME^4^ poultry	kcal	2615	2908	3200	2619	2910	3201	2556	2840	3124	2493	2770	3047	2529	2810	3091	2800
AFD^5^ Lysine	%	0.92	1.02	1.12	0.85	0.94	1.03	0.76	0.84	0.92	0.61	0.68	0.75	0.67	0.74	0.81	0.83
AFD Methionine	%	0.45	0.52	0.58	0.42	0.48	0.55	0.38	0.44	0.49	0.30	0.34	0.39	0.36	0.41	0.47	0.47
AFD Meth.+Cyst.	%	0.70	0.78	0.86	0.67	0.74	0.81	0.61	0.68	0.75	0.51	0.57	0.63	0.58	0.64	0.70	0.71
AFD Threonine	%	0.59	0.66	0.73	0.55	0.61	0.67	0.50	0.56	0.62	0.42	0.47	0.52	0.46	0.51	0.56	0.58
AFD Tryptophan	%	0.20	0.21	0.21	0.19	0.20	0.20	0.18	0.19	0.19	0.15	0.16	0.17	0.15	0.16	0.18	0.18
AFD Isoleucine	%	0.65	0.72	0.79	0.60	0.67	0.74	0.56	0.62	0.68	0.46	0.53	0.61	0.52	0.58	0.64	0.65
AFD valine	%	0.76	0.82	0.88	0.71	0.77	0.82	0.67	0.72	0.77	0.57	0.63	0.69	0.62	0.67	0.72	0.73
AFD arginine	%	1.10	1.16	1.23	1.03	1.08	1.14	0.97	1.01	1.05	0.81	0.87	0.92	0.87	0.93	0.98	1.01
**Analyzed chemical composition**																	
DM	%	87.60	88.73	88.50	88.20	89.71	89.20	88.20	89.27	89.10	87.70	88.60	88.60	88.70	89.11	89.90	89.50
Crude protein	%	19.70	19.74	21.10	18.70	19.83	20.70	18.00	18.36	19.50	14.70	16.92	16.60	16.30	20.06	18.00	18.10
Crude fat	%	5.90	8.08	9.00	6.90	6.82	8.30	5.00	5.30	6.60	4.50	5.26	4.80	4.60	6.24	7.60	4.70
Moisture	%	12.40	11.27	11.50	11.80	10.29	10.80	11.80	10.73	10.90	12.30	11.40	11.40	11.30	10.89	10.10	10.50
Calcium	%	0.85	1.07	1.10	0.95	1.07	1.15	0.91	0.89	1.02	0.33	2.96	1.03	2.47	5.59	2.93	4.32
Phosphorous	%	0.69	0.68	0.68	0.74	0.69	0.67	0.68	0.59	0.58	0.70	0.59	0.69	0.65	0.70	0.70	0.59
Sodium^6^	%		0.22	0.20		0.20	0.17	0.14	0.13	0.12	0.09	0.12	0.13	0.16	0.14		

1 Hostazym X 15,000.

2 Phyzyme XP 10,000 TPT–500 FTU.

3 Supplied per kg diet rearing: Vitamin A (retinyl-acetate), 10,000 IU; vitamin D3 (cholecalciferol), 3,500 IU; vitamin E (DL-α-tocopherol), 100 mg; vitamin K3 (menadione), 3.0 mg; vitamin B1 (thiamine), 3.0 mg; vitamin B2 (riboflavin), 6.0 mg; vitamin B6 (pyridoxine-HCL) 3.0 mg; vitamin B12 (cyanocobalamine), 20 μg; niacine, 35 mg; D-pantothenic acid, 15 mg; choline chloride, 600 mg; folic acid, 1.5 mg; biotin, 150 μg; FeSO_4_·H_2_O, 133 mg; CuSO_4_·5H_2_O, 64 mg; MnO, 190 mg; ZnSO_4_·H_2_O, 306 mg; KI, 1.2 mg; Na_2_SeO_3_, 0.7 mg. Supplied per kg diet lay: Vitamin A (retinyl-acetate), 10,000 IU; vitamin D3 (cholecalciferol), 2,000 IU; vitamin E (DL-α-tocopherol), 25 mg; vitamin K3 (menadione), 1.5 mg; vitamin B1 (thiamine), 1.0 mg; vitamin B2 (riboflavin), 3.5 mg; vitamin B6 (pyridoxine-HCL) 1.0 mg; vitamin B12 (cyanocobalamine), 15 μg; niacine, 30 mg; D-pantothenic acid, 12 mg; choline chloride, 350 mg; folic acid, 0.8 mg; biotin, 100 μg; FeSO_4_·H_2_O, 167 mg; CuSO_4_·5H_2_O, 40 mg; MnO, 100 mg; ZnSO_4_·H_2_O, 150 mg; KI, 1.0 mg; Na_2_SeO_3_, 0.22 mg.

4 Metabolizable energy, calculated according to CVB, 2016.

5 Apparent fecal digestible, calculated according to CVB, 2016.

6 Analyzed in a second batch, some missing values due to storage issues.

### Data Collection

From 0 until 20 wk of age, individual pullet BW were collected on a weekly basis. From 20 wk of age onward, individual pullet BW were collected at 24, 28, 32, and 35 wk age. Based on BW, ADG was calculated per bird. On the same day as BW measurements, total feed intake per pen was measured to calculate ADFI per hen. Finally, gain to feed (**G:F**) was calculated as ADG/ADFI. After the first egg was found at 19 wk of age, eggs were collected 3 times a day and registered per pen. Eggs were classified according to first grade eggs or second grade eggs (including broken eggs, dirty eggs, shell less eggs, double yolk eggs, floor eggs, or other).

Egg production was recorded on a daily basis to calculate lay percentage. On a weekly basis eggs were individually weighed. Egg mass was calculated as laying rate × average egg weight of first class eggs. Feed conversion ratio (**FCR**) for egg mass was calculated as: ADFI/egg mass.

On all the days that BW was measured, 1 pullet in wk 0, 2, 3, 5, 6, 8, 10, 12, 14, 15, 17, 19, 20, and 35 or 2 pullets in wk 1, 4, 7, 9, 11, 13, 16, and 18 were randomly collected per pen for dissection. Pullets were euthanized by using CO_2_ and successively weighed and bled when blood flow was sufficient, from wk 5 onward. The carcasses were not defeathered for the total body composition analysis. During dissection, weights were measured of the breast filet (pectoralis major, pectoralis minor, sternum, and clavicle), liver and lastly the abdominal fat pad. For day-old chicks also the yolk was removed to calculate yolk free body mass. In collection weeks that 2 pullets per pen were dissected, all organs, the carcass and the blood were collected from the 2 pullets and stored at −20°C. Frozen material was cut with a saw into smaller pieces and subsequently ground to create homogenous samples per experimental unit. Three subsamples of approximately 100 g of ground material were taken and freeze dried. Weight loss during freeze drying was measured and included in total DM calculations. Next, samples were analyzed for DM (ISO 6496, 1999), CF (ISO 6496, 1999), CP (ISO 6496, 1999), and ash content (ISO 6496, 1999). The weight of each body component was calculated based on the analyzed results and the dissected BW. The body composition results were checked for outliers and out of the 480 collected samples, 8 measurements of adipose tissue were removed and 5 measurements of breast weights were removed. These outliers were related to the selection of bad hens (discovered after opening them), or sampling errors.

### Statistical Analysis

Data were analyzed using pen as the experimental unit. Model assumptions for normality and equal variance of the error terms were checked by inspection of the residual plots. Data were subjected to mixed model analyses, using R version 4.1.1 (R Core Team, 2013). The following statistical model was used:

$$Y_{ij}=\mu +\alpha_{i}+B_{j}+\varepsilon_{ij}$$

where *Y_ij_* = dependent variable, *μ* = overall mean, *α_i_* = fixed effect of treatment (*i* = low, medium, and high diet density), *B_j_* = random block effect (*I* = 1–8), and *ε_ij_* = residual error. Contrasts were used to determine significant relationships for linear and quadratic effects of diet density. Data were expressed as least square (**LS**) means and effects were considered to be significant when *P* ≤ 0.05.

### Allometric Growth Analysis

To determine a multiphasic allometric relationship of one body component relative to another, an overall growth curve was established and inflection points were determined, following this function (adapted from Kwakkel et al., 1993):

(1) $$y_{x}=\alpha_{0}+\beta_{i}x_{i}+\sum_{j=1}^{n-1}\;\beta_{j+1}\left(x_{i}-\gamma_{j}\right)*I_{\left(x_{i>}\gamma_{j}\right)}\;$$

where *y_x_* = the weight of a body component (e.g., DM, CF, CP, or BW; g), *x_i_* = the weight of the body component to be compared to (e.g., CF, CP, breast weight, adipose tissue, or liver; g), *a*_0_ = intercept, *ß_i_* and *ß_j_* = allometric slope at phase *i* or *j*, respectively, *ɣ_j_* = the estimated inflection point between phase 1 and 1 + 1, *I* = 1 when *x_i_* > *ɣ_j_* and 0 otherwise, and *n* = number of phases. To allow for linear comparisons, the function was transformed on a natural logarithm (**LN**) scale, except when comparing the body CF with adipose tissue and the body CP with breast weight. Curves were fitted using a segmented regression procedure in R (Muggeo, 2008). Model parameter estimation and number of inflection points were evaluated using goodness of fit criteria. These criteria included: Pearson correlation coefficient, Durbin-Watson statistic, the Bayesian information criterion (**BIC**) and an *F* test comparing the number of inflection points ranging from 0 to 2. Initially, the model parameters and goodness of fit were determined for all data without treatment effect. The model with the number of inflection points that showed the best fit was then used to determine if adding the treatment effect resulted in a significantly different model, using an *F* test. If adding a treatment effect indeed resulted in a significantly different model (*P* ≤ 0.05), in the next step, the model parameters were determined again for each treatment separately. To compare the model parameters between treatments, a 1 sample *t* test was used.

## RESULTS

### Growth Performance in the Rearing Phase (Wk 0–17)

From wk 0 to 3 and wk 12 to 17, the ADG of pullets linearly increased with diet density (*P* < 0.05; Table 2). As a result, BW also linearly increased with diet density for most ages until wk 17 (Δ = 40.7 g in wk 17 between high and low density diet; *P* < 0.01; Table 3). The body weights exceeded the breed guide from wk 2 onward dependent on treatment, but no longer at 17 wk of age (Hy-Line, 2016). During the rearing period the body weights ranged between 93 and 117% of the breed recommendations, dependent on treatment, and during the laying period the body weights ranged between 97 and 105% of the breed recommendations. ADFI linearly decreased with higher diet density in all periods (*P* < 0.01; Table 2). As a consequence, G:F linearly increased with increasing diet density in all periods (*P* < 0.01; Table 2). Despite the reduction in ADFI in higher density diets, the energy and AFD Lys intake still linearly increased with diet density (*P* < 0.05; Table 4).

**Table 2:** Effects of diet density during rearing on ADG, ADFI, and G:F of pullets, expressed as least squares means.

	ADG, g/d					ADFI, g/d					G:F, g/d				
Period in wk	0–3	3–6	6–12	12–15	15–17	0–3	3–6	6–12	12–15	15–17	0–3	3–6	6–12	12–15	15–17
**Dietary nutrient density**															
Low	8.34	14.21	12.43	6.41	3.51	17.27	40.13	64.14	72.84	62.93	0.483	0.355	0.194	0.088	0.056
Medium	8.57	14.09	12.61	6.98	3.61	15.99	36.78	59.06	66.86	57.82	0.536	0.384	0.214	0.104	0.062
High	8.67	14.30	12.81	7.16	4.14	14.82	34.05	57.60	63.77	53.48	0.585	0.421	0.223	0.112	0.077
SEM (n = 8)	0.06	0.11	0.18	0.21	0.24	0.14	0.59	0.58	0.87	0.68	0.004	0.008	0.003	0.003	0.004
***P* value**															
Fixed effect	0.002	0.350	0.308	0.037	0.016	<0.001	<0.001	<0.001	<0.001	<0.001	<0.001	<0.001	<0.001	<0.001	<0.001
Linear effect	0.001	0.504	0.132	0.014	0.007	<0.001	<0.001	<0.001	<0.001	<0.001	<0.001	<0.001	<0.001	<0.001	<0.001
Quadratic effect	0.334	0.202	0.980	0.435	0.235	0.619	0.443	0.023	0.199	0.643	0.570	0.487	0.173	0.251	0.145

**Table 3:** Effects of diet density on body weight in gram, expressed as least squares means.

Age in wk	0	1	2	3	4	5	6	7	8	9	10	11	12
**Dietary nutrient density**													
Low	37.8	74.4	133.1	205.5	296.3	402.3	505.0	606.1	707.4	778.6	867.6	943.7	1,028.3
Medium	37.7	75.1	136.0	210.1	300.1	406.9	507.7	619.0	718.5	789.5	876.6	960.1	1,035.1
High	37.9	74.9	137.4	212.5	299.5	409.1	513.5	618.3	722.6	794.8	886.3	968.0	1,044.5
SEM (n = 8)	0.51	0.41	0.66	0.83	1.52	2.49	2.87	4.66	6.42	5.16	4.93	5.21	5.78
***P* value**													
Fixed effect	0.581	0.437	0.001	<0.001	0.187	0.170	0.113	0.082	0.113	0.091	0.054	0.010	0.176
Linear effect	0.824	0.387	<0.001	<0.001	0.151	0.069	0.044	0.058	0.046	0.034	0.017	0.003	0.068
Quadratic effect	0.316	0.344	0.335	0.303	0.245	0.685	0.651	0.204	0.572	0.645	0.950	0.485	0.855

Age in wk	13	14	15	16	17	18	19	20	24	28	32	35	

**Dietary nutrient density**													
Low	1,079.0	1,132.7	1,165.3	1,194.7	1,216.1	1,289.8	1,386.9	1,478.9	1,598.0	1,628.0	1,676.6	1,683.1	
Medium	1,092.9	1,145.7	1,180.9	1,207.4	1,231.1	1,287.2	1,385.1	1,490.2	1,626.7	1,662.0	1,723.5	1,733.7	
High	1,105.7	1,156.0	1,197.1	1,233.0	1,256.8	1,296.6	1,397.7	1,503.7	1,639.7	1,692.5	1,747.5	1,733.2	
SEM (n = 8)	6.60	6.81	6.83	5.96	8.06	9.05	11.74	12.80	0.01	0.02	0.02	0.02	
***P* value**													
Fixed effect	0.024	0.059	0.004	<0.001	0.001	0.664	0.700	0.414	0.121	0.052	0.013	0.060	
Linear effect	0.007	0.019	0.001	<0.001	<0.001	0.530	0.510	0.192	0.048	0.017	0.004	0.040	
Quadratic effect	0.947	0.859	0.970	0.306	0.483	0.523	0.608	0.947	0.645	0.934	0.534	0.203

**Table 4:** Effects of diet density during rearing on energy and AFD^1^ Lys intake of pullets, expressed as least squares means.

	Energy intake, kcal/d					AFD^1^ Lys intake, mg/d				
Period in wk	0–3	3–6	6–12	12–15	15–17	0–3	3–6	6–12	12–15	15–17
**Dietary nutrient density**										
Low	44.6	103.5	161.6	179.2	157.3	156.9	332.2	473.3	439.2	407.8
Medium	45.8	105.4	165.4	182.8	160.6	161.5	338.3	484.3	448.0	416.3
High	46.7	107.4	177.4	191.8	163.4	164.7	344.5	519.6	470.0	423.5
SEM (n = 8)	0.4	1.7	1.7	2.3	1.8	1.4	5.4	4.9	5.7	4.8
***P* value**										
Fixed effect	<0.001	0.036	<0.001	0.005	0.096	<0.001	0.036	<0.001	0.005	0.095
Linear effect	<0.001	0.011	<0.001	0.002	0.034	<0.001	0.011	<0.001	0.002	0.033
Quadratic effect	0.541	0.991	0.060	0.359	0.919	0.541	0.991	0.060	0.359	0.919

1 Apparent fecal digestible, calculated according to CVB, 2016.

### Body Composition in the Rearing Phase (Wk 0–17)

Ash, CP and CF, expressed as percentage of BW, sum to nearly the full DM value, expressed as percentage of BW. Minor differences are expected to be caused by analytical error. Body DM quadratically increased with diet density in wk 2, 3, 6, 14, and 15 (*P* < 0.05; Table 5), with a quadratic tendency in wk 14 (*P* = 0.057). Similar to body DM, body CF quadratically increased with diet density in wk 2, 3, 6, 14, 15, and 17 (Δ = 2.32% in wk 17 between high and low density diet; *P* < 0.05; Table 6). Body CP was not influenced by diet density, except for a linear increase with diet density in wk 8 (*P* = 0.03; Table 7). Lastly, body ash was not affected by treatment, except for a linear increase with diet density in wk 10 (*P* = 0.04; Table 8) and a quadratic reduction with diet density in wk 17 (*P* = 0.04).

**Table 5:** Effects of diet density on body DM in % of BW, expressed as least squares means.

Age in wk	0	2	3	5	6	8	10	12	14	15	17	19	20	35
**Dietary nutrient density**														
Low	17.09	25.65	26.16	26.69	27.33	29.29	29.58	31.57	32.84	33.22	35.16	36.68	38.99	40.58
Medium	17.27	27.71	26.93	27.39	28.91	29.59	30.23	31.93	34.24	35.47	35.96	37.54	40.22	40.81
High	17.20	27.56	28.50	27.05	28.91	30.47	31.03	32.56	34.49	36.38	36.48	36.27	38.08	40.38
SEM (n = 8)	0.50	0.35	0.47	0.28	0.34	0.65	0.41	0.46	0.55	0.80	0.62	0.64	0.74	0.59
***P* value**														
Fixed effect	0.962	<0.01	<0.01	0.235	0.007	0.389	0.077	0.333	0.058	0.032	0.346	0.378	0.142	0.877
Linear effect	0.919	0.671	0.001	0.397	0.993	0.316	0.191	0.345	0.715	0.418	0.562	0.179	0.054	0.615
Quadratic effect	0.800	<0.01	<0.01	0.139	0.002	0.349	0.057	0.253	0.020	0.013	0.185	0.775	0.855	0.984

**Table 6:** Effects of diet density on body crude fat in % of BW, expressed as least squares means.

Age in wk	0	2	3	5	6	8	10	12	14	15	17	19	20	35
**Dietary nutrient density**														
Low	3.50	4.75	4.19	3.59	3.25	5.19	5.34	6.27	6.65	6.84	7.19	11.07	12.93	16.49
Medium	3.46	5.66	4.23	3.62	4.38	5.54	6.12	6.52	8.12	8.55	8.58	11.13	14.02	16.18
High	3.46	5.87	5.72	3.70	4.88	5.82	6.01	7.05	8.24	10.08	9.51	10.34	12.07	15.81
SEM (n = 8)	0.27	0.23	0.26	0.26	0.28	0.41	0.41	0.47	0.42	0.72	0.60	0.67	0.69	0.56
***P* value**														
Fixed effect	0.989	0.003	0.001	0.947	0.001	0.574	0.327	0.511	0.034	0.009	0.023	0.662	0.043	0.703
Linear effect	0.983	0.467	0.001	0.819	0.169	0.639	0.840	0.439	0.839	0.107	0.233	0.421	0.014	0.649
Quadratic effect	0.886	0.001	0.017	0.819	<0.01	0.352	0.145	0.393	0.011	0.006	0.012	0.692	0.855	0.488

**Table 7:** Effects of diet density on body CP in % of BW, expressed as least squares means.

Age in wk	0	2	3	5	6	8	10	12	14	15	17	19	20	35
**Dietary nutrient density**														
Low	11.20	16.52	17.45	18.00	19.09	18.97	19.66	20.67	21.41	21.08	22.20	20.32	21.29	19.68
Medium	11.29	16.95	17.52	18.28	18.73	19.10	19.54	20.80	21.16	21.37	22.27	20.38	21.29	19.66
High	11.26	16.79	17.55	17.56	18.40	19.85	19.98	20.56	21.25	21.12	21.72	20.37	20.07	19.64
SEM (n = 8)	0.28	0.25	0.33	0.29	0.38	0.26	0.31	0.34	0.35	0.35	0.39	0.47	0.41	0.32
***P* value**														
Fixed effect	0.967	0.277	0.927	0.251	0.461	0.036	0.470	0.759	0.852	0.670	0.545	0.991	0.071	0.996
Linear effect	0.936	0.537	0.894	0.105	0.550	0.032	0.244	0.466	0.841	0.494	0.319	0.986	0.045	0.953
Quadratic effect	0.810	0.143	0.721	0.823	0.280	0.117	0.739	0.985	0.603	0.604	0.662	0.893	0.227	0.950

**Table 8:** Effects of diet density on body ash in % of BW, expressed as least squares means.

Age in wk	0	2	3	5	6	8	10	12	14	15	17	19	20	35
**Dietary nutrient density**														
Low	1.37	2.69	2.86	3.07	3.07	3.02	3.17	3.39	3.13	3.30	4.12	3.22	3.36	3.15
Medium	1.39	2.90	3.02	2.97	3.28	3.04	2.90	3.29	2.98	3.57	3.45	3.29	3.45	3.25
High	1.32	2.71	2.93	3.04	3.19	3.06	3.46	3.49	3.18	3.55	3.41	2.99	3.46	3.22
SEM (n = 8)	0.05	0.08	0.11	0.17	0.09	0.14	0.18	0.13	0.21	0.19	0.25	0.28	0.23	0.17
***P* value**														
Fixed effect	0.538	0.171	0.543	0.840	0.256	0.982	0.121	0.534	0.768	0.376	0.109	0.720	0.936	0.869
Linear effect	0.278	0.117	0.528	0.678	0.474	0.930	0.043	0.271	0.507	0.925	0.901	0.445	0.962	0.887
Quadratic effect	0.790	0.254	0.370	0.684	0.139	0.868	0.962	0.993	0.821	0.173	0.039	0.793	0.723	0.616

### Growth and Production Performance in the Laying Phase (Wk 17–35)

From wk 17 to 20, ADG of hens fed higher density diets during rearing was linearly decreased (*P* < 0.05; Table 9). In that same age period, ADFI, energy intake, and AFD Lys intake of hens fed the higher diet density during rearing were linearly decreased (*P* < 0.05; Table 9). In wk 32 to 35, ADG of hens fed the high density diets during rearing was linearly decreased (*P* < 0.05; Table 9). Only hens fed the highest density diets were in a negative energy balance in that time period, with a negative ADG of −0.72 g. ADFI, energy intake, and AFD Lys intake were not affected by diet density during rearing in any time period between wk 17 and 35. The BW of laying hens fed higher diet density during rearing was linearly increased in wk 24, 28, 32, and 35 (*P* < 0.05; Table 3). Egg production, egg weight, egg mass and FCR for egg mass until wk 35 was not affected by diet density during rearing (Table 10).

**Table 9:** Effects of pullet diet density on ADG, ADFI, and laying rate, expressed as least squares means.

Period in wk	17–20	20–24	24–28	28–32	32–35	17–20	20–24	24–28	28–32	32–35
	**ADG, g/d**					**ADFI, g/d**				
**Dietary nutrient density**										
Low	12.48	4.57	1.07	2.06	0.33	78.53	94.22	109.90	115.81	120.71
Medium	11.40	4.87	1.26	2.20	0.51	75.50	96.76	110.96	116.43	123.14
High	10.78	4.86	1.88	1.97	−0.72	72.81	98.39	109.66	119.27	122.57
SEM (n = 8)	0.35	0.44	0.29	0.34	0.33	1.12	1.14	1.37	2.05	1.95
***P* value**										
Fixed effect	0.013	0.855	0.134	0.828	0.038	0.010	0.063	0.756	0.464	0.604
Linear effect	0.004	0.638	0.058	0.809	0.039	0.003	0.022	0.901	0.252	0.484
Quadratic effect	0.598	0.758	0.534	0.589	0.097	0.901	0.750	0.466	0.665	0.501
	**Energy intake, kcal/d**					**AFD Lys intake, mg/d**				
**Dietary nutrient density**										
Low	219.9	263.8	307.7	324.3	338.0	651.8	782.0	912.1	961.2	1001.9
Medium	211.4	270.9	310.7	326.0	344.8	626.7	803.1	920.9	966.4	1022.1
High	203.9	275.5	307.1	333.9	343.2	604.4	816.6	910.2	989.9	890.4
SEM (n = 8)	3.1	3.2	3.8	5.7	5.5	9.3	9.5	11.3	17.0	74.9
***P* value**										
Fixed effect	0.010	0.063	0.756	0.464	0.604	0.010	0.063	0.756	0.464	0.414
Linear effect	0.003	0.330	0.498	0.343	0.827	0.003	0.330	0.498	0.343	0.224
Quadratic effect	0.901	0.031	0.797	0.430	0.341	0.901	0.031	0.797	0.430	0.618

**Table 10:** Effects of pullet diet density on egg weight, egg mass, and FCR for egg mass, expressed as least squares means.

Period in wk	17–20	20–24	24–28	28–32	32–35	17–20	20–24	24–28	28–32	32–35
	**Laying rate, %**					**Egg weight, g**				
**Dietary nutrient density**										
Low	80.66	93.70	94.65	96.53	81.45	44.44	51.14	56.06	58.91	61.52
Medium	80.62	95.10	94.97	96.62	80.39	44.29	51.95	57.05	59.70	61.78
High	79.65	91.68	94.01	95.40	80.61	42.88	51.89	56.97	60.14	61.80
SEM (n = 8)	1.82	1.34	1.50	0.90	0.88	0.91	0.29	0.37	0.41	0.35
***P* value**										
Fixed effect	0.894	0.229	0.875	0.548	0.612	0.317	0.076	0.131	0.141	0.813
Linear effect	0.688	0.306	0.729	0.374	0.476	0.176	0.057	0.094	0.055	0.562
Quadratic effect	0.828	0.164	0.708	0.559	0.499	0.537	0.187	0.243	0.737	0.777
	**Egg mass, g**					**FCR for egg mass**				
**Dietary nutrient density**										
Low	41.23	52.54	55.75	56.86	45.23	2.37	2.09	2.08	2.13	2.40
Medium	42.76	54.26	55.77	57.68	45.43	2.32	2.05	2.09	2.14	2.38
High	41.34	52.22	56.53	58.34	45.01	2.39	2.11	2.11	2.09	2.44
SEM (n = 8)	0.93	0.82	0.86	0.88	0.63	0.07	0.03	0.03	0.04	0.04
***P* value**										
Fixed effect	0.425	0.167	0.718	0.464	0.879	0.774	0.227	0.827	0.546	0.608
Linear effect	0.926	0.770	0.483	0.224	0.801	0.829	0.675	0.551	0.413	0.504
Quadratic effect	0.199	0.066	0.696	0.938	0.675	0.502	0.096	0.912	0.443	0.466

LSmeans within a column and factor lacking a common superscript differ (*P* < 0.05).

### Body Composition in the Laying Phase (Wk 18–35)

Body CP and CF were linearly affected by rearing diet density only in wk 20. The body CF was higher of hens fed the medium density diets during rearing, compared to the hens fed the high density diets during rearing (*P* < 0.05; Table 6). Body DM and body crude ash were not affected by rearing diet density.

No effect of diet density on breast weight percentage was found (Table 11). Abdominal fat pad weight (measurable from wk 2 onward) increased linearly with diet density in wk 2, 3, 6, 13, 14, 15, and 17 (Δ = 0.50% in wk 17 between high and low density diets; *P* < 0.01; Table 12).

**Table 11:** Effects of diet density on relative breast in %, expressed as least squares means.

Age in wk	1	2	3	4	5	6	7	8	9	10	11
**Dietary nutrient density**											
Low	8.79	11.94	12.91	13.94	14.79	15.51	15.64	16.16	16.81	17.69	18.41
Medium	8.93	11.61	13.05	14.06	14.31	15.26	15.53	16.34	16.81	17.23	17.41
High	8.62	12.14	13.00	13.45	14.38	15.48	16.25	16.82	16.82	16.89	17.44
SEM (n = 8)	0.36	0.24	0.28	0.28	0.23	0.34	0.37	0.27	0.32	0.28	0.41
***P* value**											
Fixed effect	0.790	0.252	0.939	0.188	0.306	0.844	0.331	0.235	0.999	0.078	0.178
Linear effect	0.695	0.536	0.825	0.160	0.222	0.948	0.244	0.104	0.970	0.027	0.106
Quadratic effect	0.581	0.126	0.787	0.231	0.319	0.569	0.354	0.668	0.994	0.832	0.315

Age in wk	12	13	14	15	16	17	18	19	20	35	

**Dietary nutrient density**											
Low	17.45	17.78	19.08	19.03	19.57	19.47	19.98	18.31	17.87	15.85	
Medium	18.17	18.10	18.62	18.79	19.94	19.91	19.47	18.73	18.53	15.96	
High	18.28	18.55	18.85	18.60	18.89	19.85	19.36	18.72	17.49	15.83	
SEM (n = 8)	0.32	0.25	0.34	0.24	0.40	0.38	0.33	0.27	0.27	0.23	
***P* value**											
Fixed effect	0.181	0.093	0.638	0.381	0.161	0.448	0.354	0.342	0.049	0.912	
Linear effect	0.092	0.033	0.646	0.174	0.215	0.309	0.183	0.213	0.340	0.693	
Quadratic effect	0.447	0.842	0.414	0.915	0.137	0.457	0.608	0.446	0.023	0.881	

LSmeans within a column and factor lacking a common superscript differ (*P* < 0.05).

**Table 12:** Effects of diet density on relative abdominal fat in %, expressed as least squares means.

Age in wk	2	3	4	5	6	7	8	9	10	11	12
**Dietary nutrient density**											
Low	0.18	0.24	0.17	0.18	0.11	0.39	0.39	0.34	0.41	0.52	0.58
Medium	0.33	0.29	0.28	0.19	0.25	0.51	0.50	0.42	0.53	0.59	0.58
High	0.31	0.45	0.28	0.19	0.40	0.62	0.47	0.59	0.61	0.72	0.73
SEM (n = 8)	0.03	0.04	0.05	0.04	0.05	0.09	0.08	0.10	0.09	0.12	0.10
***P* value**											
Fixed effect	0.010	0.010	0.175	0.952	0.001	0.143	0.633	0.189	0.257	0.541	0.439
Linear effect	0.013	0.003	0.103	0.797	0.000	0.052	0.512	0.077	0.107	0.282	0.264
Quadratic effect	0.041	0.364	0.394	0.875	0.850	0.956	0.493	0.756	0.852	0.854	0.504

Age in wk	12	13	14	15	16	17	18	19	20	35	

**Dietary nutrient density**											
Low	0.58	0.62	0.59	0.66	1.11	0.86	1.22	2.02	2.23	2.97	
Medium	0.58	0.58	1.18	1.18	1.29	0.92	1.60	1.76	2.28	3.04	
High	0.73	1.06	0.88	1.29	1.07	1.36	1.56	1.65	2.18	2.75	
SEM (n = 8)	0.10	0.14	0.09	0.15	0.23	0.11	0.25	0.19	0.18	0.22	
***P* value**											
Fixed effect	0.439	0.054	0.002	0.021	0.716	0.002	0.521	0.416	0.917	0.521	
Linear effect	0.264	0.042	0.044	0.009	0.883	0.001	0.353	0.203	0.837	0.281	
Quadratic effect	0.504	0.160	0.002	0.293	0.426	0.074	0.515	0.775	0.723	0.745	

### Allometric Growth of Liver

Modeling the LN of BW × liver (g) showed the best model fit with 2 inflection points (BIC −347 vs. −498, for respectively 1 or 2 inflection points) and a significant treatment effect (*P* < 0.05; Table 13). The first slope (*ß_i_* from Equation 1) was significantly higher for pullets fed the high density diets compared with the medium density diets, with the lowest slope of the pullets being those fed the low density diets. The second slope (*ß_j_* from Equation 1) was significantly higher for the pullets fed the high density diets compared to the medium density diets.

**Table 13:** Functional specifications, coefficients, and goodness-of-fit criteria of the segmented regression models^1^ describing the LN of body weight × LN liver weight of pullets.

	1 Inflection point			2 Inflection points			1 Inflection point			2 Inflection points		
Parameters^1^	Estimate	SEM	*P* value	Estimate	SEM	*P* value	Estimate	SEM	*P* value	Estimate	SEM	*P* value
	**Overall model**						**Low diet density**					
ɣ_i_	1.20	0.11	-	2.40	0.05	-	1.30	0.18	-	2.31^a^	0.09	-
α_0_	3.54	0.04	<0.01	3.46	0.02	<0.01	3.56	0.06	<0.01	3.49^ab^	0.05	<0.01
ß_i_	0.78	0.05	<0.01	0.96	0.03	<0.01	0.77	0.08	<0.01	0.92^ab^	0.04	<0.01
ß_j_	1.24	0.05		1.64	0.02		1.26	0.09		1.60^a^	0.07	
ɣ_k_	-	-	-	3.14	0.02	-	-	-	-	3.22^a^	0.04	-
ß_k_	-	-	-	−0.10	0.07	-	-	-	-	−0.17	−1.10	-
R^2^	0.983			0.985			0.983			0.986		
R^2^ -adjusted	0.971			0.979			0.971			0.979		
DW^2^	0.71			0.86			0.84			1.12		
BIC^3^	−347			−498			−99			−148		
*P* value^4^ number of inflection points	<0.01			<0.01			<0.01			0.00		
*P* value^4^ treatment effect	-			<0.01			-			-		

Parameters^1^	**Medium diet density**						**High diet density**					
ɣ_i_	1.18	0.20	-	2.68^b^	0.07	-	1.13	0.25	-	2.54^a^	0.09	-
α_0_	3.56	0.06	<0.01	3.44^a^	0.04	<0.01	3.51	0.07	<0.01	3.41^b^	0.05	<0.01
ß_i_	0.77	0.10	<0.01	1.00^a^	0.02	<0.01	0.81	0.11	<0.01	1.01^b^	0.03	<0.01
ß_j_	1.23	0.10		2.03^b^	0.20		1.22	0.12		1.72^ab^	0.11	
ɣ_k_	-	-	-	3.09^b^	0.028	-	-	-	-	3.123^a^	0.033	-
ß_k_	-	-	-	−0.46	0.22	-	-	-	-	−0.15	0.15	-
R^2^	0.984			0.986			0.985			0.987		
R^2^ -adjusted	0.972			0.980			0.971			0.979		
DW^2^	0.76			1.05			0.78			0.74		
BIC^3^	−107			−157			−98			−142		
*P* value^4^ number of inflection points	<0.01			0.00			<0.01			0.00		
*P* value^4^ treatment effect	-			-			-			-		

1 $y_{x}=\alpha_{0}+\beta_{i}x_{i}+\sum_{j=1}^{n-1}\beta_{j+1}\left(x_{i}-\gamma_{j}\right)*I_{\left(x_{i>}\gamma_{j}\right)}$, in which *ɣ_ik_* is the inflection point in phase *i* to *j, α*_0_ is the intercept, *ß_ijk_* is the allometric slope at phase *i, j*, or *k*.

2 Durbin-Watson statistic; values range from 0 to 4, values of 2 indicate no autocorrelation, value less than 2 indicates a positive autocorrelation, a value greater than 2 a negative autocorrelation.

3 Bayesian information criterion, lower values indicate a better model fit.

4 The *P* value obtained with an *F* test. *P* values compare the linear model with the 1 inflection point model, and the 1 inflection point model with the 2 inflection point model. If the *F* test was significant for treatment effect (*P* < 0.05), the model parameters between treatments were compared using a pairwise *t* test. Values with unique superscripts for the same parameter were significantly different (*P* < 0.05).

### Allometric Growth of Body Chemical Composition

Modeling the LN of body DM × body CP (g; Table 14) showed that the best model fit has 1 inflection point (BIC = −954 for both the 1 and 2 inflection point model in the overall model; for all individual treatment models, lower BIC values for the 1 inflection point model vs. the 2 inflection point model were found, data not shown). Dietary treatment significantly influenced the model, but only resulted in a slightly higher slope in the first phase (*ß_i_*, from Equation 1) of the pullets fed the medium diet compared to the high density diet.

**Table 14:** Functional specifications, coefficients, and goodness-of-fit criteria of the segmented regression models^1^ describing the LN of body dry matter × body crude protein of pullets.

	1 Inflection point			2 Inflection points			1 Inflection point			1 Inflection point			1 Inflection point		
Parameters^1^	Estimate	SEM	*P* value	Estimate	SEM	*P* value	Estimate	SEM	*P* value	Estimate	SEM	*P* value	Estimate	SEM	*P* value
Treatments	Overall model						Low diet density			Medium diet density			High diet density		

ɣ_i_	5.89	0.02	-	2.18	0.20	-	5.85	0.04	-	5.86	0.04	-	5.94	0.03	-
α_0_	−0.44	0.01	<0.01	5.86	0.02	<0.01	−0.43	0.02	<0.01	−0.45	0.02	<0.01	−0.45	0.02	<0.01
ß_i_	1.00	0.00	<0.01	−0.13	0.17	<0.01	1.005	0.00	<0.01	1.003	0.00	<0.01	1.000	0.00	<0.01
ß_j_	0.57	0.03		0.85	0.09		0.57	0.04		0.62	0.04		0.52	0.05	
ɣ_k_	-	-	-	5.86	0.02	-	-	-	-	-	-	-	-	-	-
ß_k_	-	-	-	0.41	0.03	-	-	-	-	-	-	-	-	-	-
R^2^	0.999			0.999			0.999			0.999			0.999		
R^2^ -adjusted	0.999			0.999			0.999			0.999			0.999		
DW^2^	1.75			1.80			1.76			1.78			1.67		
BIC^3^	−954			−954			−313			−317			−322		
*P* value^4^ number of inflection points	<0.01			<0.01			<0.01			<0.01			<0.01		
*P* value^4^ treatment effect	<0.01						-			-			-		

1 $y_{x}=\alpha_{0}+\beta_{i}x_{i}+\sum_{j=1}^{n-1}\beta_{j+1}\left(x_{i}-\gamma_{j}\right)*I_{\left(x_{i>}\gamma_{j}\right)}$, in which *ɣ_ik_* is the inflection point in phase *i* to *j, α*_0_ is the intercept, *ß_ijk_* is the allometric slope at phase *i, j*, or *k*.

2 Durbin-Watson statistic; values range from 0 to 4, values of 2 indicate no autocorrelation, value less than 2 indicates a positive autocorrelation, a value greater than 2 a negative autocorrelation.

3 Bayesian information criterion, lower values indicate a better model fit.

4 The *P* value obtained with an *F* test. *P* values compare the linear model with the 1 inflection point model, and the 1 inflection point model with the 2 inflection point model. If the *F* test was significant for treatment effect (*P* < 0.05), the model parameters between treatments were compared using a pairwise *t* test. Values with unique superscripts in a row were significantly different (*P* < 0.05).

Modeling the LN of body DM × body CF (g) showed that the best model fit had 2 inflection points (BIC = −143 vs. −165, for respectively 1 or 2 inflection points) and a significant treatment effect (*P* < 0.05; Table 15). The first inflection point (*ɣ_i_*, from Equation 1) was significantly lower for pullets fed the high density diets than for those fed the medium diets. Also the second inflection point (*ɣ_k_*, from Equation 1) was significantly lower for the pullets fed the high density diets compared to both the low and medium density diets. At the same time, the slope of the second phase (*ßj* from Equation 1) was significantly lower with the pullets fed the high density diets compared to the pullets fed the medium diets. No other model parameters were significantly influenced by dietary treatment.

**Table 15:** Functional specifications, coefficients, and goodness-of-fit criteria of the segmented regression models^1^ describing the LN of body dry matter × body crude fat of pullets.

	1 Inflection point			2 Inflection points			1 Inflection point			2 Inflection points		
Parameters^1^	Estimate	SEM	*P* value	Estimate	SEM	*P* value	Estimate	SEM	*P* value	Estimate	SEM	*P* value
	**Overall model**						**Low diet density**					
ɣ_i_	5.14	0.06	-	4.91	0.09	-	5.02	0.10	-	4.91^ab^	0.12	-
α_0_	−1.33	0.06	<0.01	−1.32	0.05	<0.01	−1.20	0.11	<0.01	−1.23	0.10	<0.01
ß_i_	0.88	0.02	<0.01	0.87	0.06	<0.01	0.83	0.03	<0.01	0.84	0.03	<0.01
ß_j_	1.62	0.05		1.39	0.02		1.62	0.08		1.43^ab^	0.07	
ɣ_k_	-	-	-	6.00	0.05	-	-	-	-	6.05^ab^	0.08	-
ß_k_	-	-	-	1.65	0.15	-	-	-	-	1.89	1.05	-
R^2^	0.983			0.985			0.983			0.986		
R^2^ -adjusted	0.983			0.984			0.983			0.985		
DW^2^	1.61			1.73			1.47			1.74		
BIC^3^	−143			−165			−37			−47		
*P* value^4^ number of inflection points	<0.01			<0.01			<0.01			0.00		
*P* value^4^ treatment effect	-			<0.01			-			-		

Parameters^1^	**Medium diet density**						**High diet density**					
ɣ_i_	5.10	0.11	-	4.86	0.14	-	5.28	0.10	-	2.68	0.64	-
α_0_	−1.38	0.10	<0.01	−1.35	0.11	<0.01	−1.39	0.10	<0.01	−2.37	0.94	<0.01
ß_i_	0.89	0.03	<0.01	0.88	0.03	<0.01	0.92	0.02	<0.01	1.40	0.50	<0.01
ß_j_	1.61	0.07		1.57	0.08		1.62	0.08		0.74	0.51	
ɣ_k_	-	-	-	6.05	0.107	-	-	-	-	5.12	0.089	-
ß_k_	-	-	-	1.23	0.28	-	-	-	-	2.28	0.09	-
R^2^	0.984			0.986			0.985			0.987		
R^2^ -adjusted	0.984			0.985			0.985			0.986		
DW^2^	1.66			1.78			1.48			1.67		
BIC^3^	−42			−42			−51			−55		
*P* value^4^ number of inflection points	<0.01			0.02			<0.01			0.00		
*P* value^4^ treatment effect	-			-			-			-		

1 $y_{x}=\alpha_{0}+\beta_{i}x_{i}+\sum_{j=1}^{n-1}\beta_{j+1}\left(x_{i}-\gamma_{j}\right)*I_{\left(x_{i>}\gamma_{j}\right)}$, in which *ɣ_ik_* is the inflection point in phase *i* to *j, α*_0_ is the intercept, *ß_ijk_* is the allometric slope at phase *i, j*, or *k*.

2 Durbin-Watson statistic; values range from 0 to 4, values of 2 indicate no autocorrelation, value less than 2 indicates a positive autocorrelation, a value greater than 2 a negative autocorrelation.

3 Bayesian information criterion, lower values indicate a better model fit.

4 The *P* value obtained with an *F* test. *P* values compare the linear model with the 1 inflection point model, and the 1 inflection point model with the 2 inflection point model. If the *F* test was significant for treatment effect (*P* < 0.05), the model parameters between treatments were compared using a pairwise *t* test. Values with unique superscripts for the same parameter were significantly different (*P* < 0.05).

Modeling the LN of body CP × body CF (g) showed that the best model fit has 2 inflection points (BIC = 53 vs. 42, for respectively 1 or 2 inflection points) and a significant treatment effect (*P* < 0.05; Table 16). The first and second inflection points (*ɣ_i_* and *ɣ_k_*, from Equation 1) were significantly lower for pullets fed the high density diets compared to the medium and low density diet. The slope (*ß_i_* from Equation 1) of the pullets fed the high density in the first phase was significantly higher, whereas the slope in the second phase (*ß_j_* from Equation 1) was significantly lower compared to the pullets fed the medium and low density diets. The third slope (*ß_k_* from Equation 1) did not significantly differ.

**Table 16:** Functional specifications, coefficients, and goodness-of-fit criteria of the segmented regression models^1^ describing the LN of body crude protein × body crude fat of pullets.

	1 Inflection point			2 Inflection points			1 Inflection point			2 Inflection points				
Parameters^1^	Estimate	SEM	*P* value	Estimate	SEM	*P* value	Estimate	SEM	*P* value	Estimate	SEM		*P* value	
	**Overall model**						**Low diet density**							
ɣ_i_	4.74	0.07	-	4.57	0.13	-	4.64	0.12	-	4.57^ab^	0.16		-	
α_0_	−0.91	0.08	<0.01	5.43	0.06	<0.01	−0.80	0.13	<0.01	−0.85^ab^	0.12		<0.01	
ß_i_	0.87	0.02	<0.01	−0.93	0.07	<0.01	0.81	0.04	<0.01	0.82^ab^	0.03		<0.01	
ß_j_	1.84	0.08		0.87	0.02		1.83	0.13		1.58^a^	0.16			
ɣ_k_	-	-	-	5.43	0.06	-	-	-	-	5.51^ab^	0.07		-	
ß_k_	-	-	-	2.01	0.26	-	-	-	-	2.43	1.60		-	
R^2^	0.968			0.970			0.970			0.974				
R^2^ -adjusted	0.968			0.970			0.969			0.972				
DW^2^	1.52			1.69			1.18			1.54				
BIC^3^	53			42			25			20				
*P* value^4^ number of inflection points	<0.01			<0.01			<0.01			0.00				
*P* value^4^ treatment effect	-			<0.01			-			-				

Parameters^1^	**Medium diet density**						**High diet density**							
ɣ_i_	4.71	0.12	-	4.33^b^	0.24	-	4.90	0.10	-	3.50^c^	0.15		-	
α_0_	−0.96	0.13	<0.01	−0.93^b^	0.14	<0.01	−0.97	0.12	<0.01	−1.33^c^	0.16		<0.01	
ß_i_	0.88	0.04	<0.01	0.86^b^	0.04	<0.01	0.91	0.03	<0.01	1.09^c^	0.07		<0.01	
ß_j_	1.83	0.12		1.39^a^	0.15		1.86	0.13		0.13^b^		0.32		
ɣ_k_	-	-	-	5.42^b^	0.086	-	-	-	-	4.215^c^		0.123		-
ß_k_	-	-	-	1.75	0.38	-	-	-	-	2.56		0.32		-
R^2^	0.970			0.973			0.976			0.975				
R^2^ -adjusted	0.969			0.972			0.971			0.973				
DW^2^	1.52			1.80			1.64			1.83				
BIC^3^	27			26			15			13				
*P* value^4^ number of inflection points	<0.01			0.01			<0.01			0.00				
*P* value^4^ treatment effect	-			-			-			-				

1 $y_{x}=\alpha_{0}+\beta_{i}x_{i}+\sum_{j=1}^{n-1}\beta_{j+1}\left(x_{i}-\gamma_{j}\right)*I_{\left(x_{i>}\gamma_{j}\right)}$, in which *ɣ_ik_* is the inflection point in phase *i* to *j, α*_0_ is the intercept, *ß_ijk_* is the allometric slope at phase *i, j*, or *k*.

2 Durbin-Watson statistic; values range from 0 to 4, values of 2 indicate no autocorrelation, value less than 2 indicates a positive autocorrelation, a value greater than 2 a negative autocorrelation.

3 Bayesian information criterion, lower values indicate a better model fit.

4 The *P* value obtained with an *F* test. *P* values compare the linear model with the 1 inflection point model, and the 1 inflection point model with the 2 inflection point model. If the *F* test was significant for treatment effect (*P* < 0.05), the model parameters between treatments were compared using a pairwise *t* test. Values with unique superscripts for the same parameter were significantly different (*P* < 0.05).

## DISCUSSION

First, the growth performance and feed and nutrient intake during the rearing phase and then the laying phase will be discussed. Then, the body composition will be discussed and lastly the allometric growth curves of the liver and chemical body composition (DM, CP, and CF) will be discussed. Before the final conclusion, the study limitations and practical implications are given.

### Growth Performance

The energy and AFD Lys intake linearly increased in all age periods during which pullets were fed a higher diet density, despite a linear decrease in ADFI. So the hens fed the higher diet density reduced ADFI, but the reduction was not to the extent that an equal energy and AFD Lys intake was reached between treatments. This resulted in increased growth and BW, with a 40.7 g higher BW of pullets fed the high compared to the low density diets at the end of the rearing phase. This difference was caused mainly by additional body fat but not protein deposition. Between the rearing and laying phase, there was a short-term transition period in wk 17 to 20, in which the pullets fed the high density diets maintained a lower ADFI, as they also had during the rearing period. Since all hens were fed the same diet density from wk 17 onward, the lower ADFI of hens fed a higher rearing density diet resulted in a linear decrease in energy and AFD Lys intake in these weeks. Hens try to standardize their energy intake during the laying period when provided different diet densities (van Krimpen et al., 2009). From the current trial it seems, however, that hens could not adapt their ADFI capacity to dietary energy changes quickly, and therefore the hens fed the previously high density diets had a lower energy and AFD Lys intake for a short 3 wk period of time.

The fluctuation in ADFI between weeks could also be caused by the relative difference in nutrient density that was fed from the prelay to lay period. For the hens fed the low and medium density diets during rearing, the switch to the common lay diet resulted in a relative increase in nutrient concentration of energy and amino acids. For the hens fed the high density diets during rearing, the nutrient density remained relatively constant. The hens fed the low and medium density rearing diets might have needed some time to adapt to the sudden increase in nutrient availability. In the following period from wk 20 to 24, the ADFI was reversed and hens fed the higher density rearing diets linearly increased ADFI, without significantly impacting energy or AFD Lys intake. After this period, the ADFI in wk 24 to 35 was similar for all treatments and not affected by rearing diet.

The relative lower ADFI in wk 17 to 20 resulted in a linear decrease in energy intake, AFD Lys intake and ADG in hens fed the higher diet density during rearing. This probably influenced the disappearance of a significant BW effect in wk 18, 19, and 20. Interestingly, in wk 24, 28, 32, and 35, BW of the hens fed a higher density diet during rearing linearly increased again, with a 50 g heavier BW of hens fed the high density rearing diet compared to the hens fed the low density rearing diets in wk 35. From wk 32 to 35, only the hens fed a high density rearing diet were in a negative energy balance, that is, energy expenditure exceeded energy intake and resulted in body mass loss, as indicated by a linear reduced ADG. It is generally believed that smaller pullets and hens have a higher risk to get into a negative energy balance during peak production, due to smaller body reserves and potentially a lower feed intake capacity (Miles and Jacob, 2000). The current study shows the opposite, with the pullets and hens with the highest BW and fat pad energy reserves at the end of the rearing phase in a negative energy balance in wk 32 to 35, whereas the pullets and hens with the lowest BW and fat pad reserves were not in a negative energy balance during this study. Body CF but not body CP was significantly different at the end of the rearing phase, which could hint at an influencing mechanism of body CF level on growth efficiency. An excess of body fat in mammals is known to negatively influence reproduction, mediated through adipokine hormones which are produced by adipose tissue (Sirotkin et al., 2018; Mintziori et al., 2020). Also in poultry, recent studies into adipokines have shown their influencing effect on lipid deposition, body weight and feed intake (Mellouk et al., 2018a). Adipokine production might be influenced by dietary factors, such as oil type, so are a promising metabolite to include in future studies (Mellouk et al., 2018b).

Current results indicate that differences in BW at the end of the rearing phase, obtained by feeding different diet densities, were maintained during the early peak production phase despite switching to a common lay feed for all treatments. Feeding different diet densities during the rearing period did not affect egg production performance (laying rate, egg weight, egg mass, FCR for egg mass) during the early peak production phase. The underlying body composition will be discussed next.

### Body Composition

Neither body CP nor breast percentage was influenced by diet in our study. The abdominal fat pad weight, relative to BW, as well as the body CF percentage linearly increased with diet density in pullets and laying hens, already from wk 2 onward until wk 6 and from wk 14 until the end of the rearing period in wk 17. Body CF percentage in the current study was around 5% lower in wk 17 than the white hens studied by Alves et al. (2019), although the BW and diets were comparable in energy and protein to our medium and high density diets. This difference could be caused by the a difference in sampling method; Alves et al. (2019) used feather free body, whereas in our study, feathers were included in the analysis. This might result in a relatively higher protein contribution and a dilution of CF. Indeed, the body CP percentage was 4% higher in our study in wk 17. Using average feather weight, DM and CP content, calculations (not shown) indicate that defeathering would have account for an average of 5% reduction in body CP percentage and a 1% increase in body CF percentage. The hens in our study were therefore very close to the body CP as measured by Alves et al. (2019), but higher in body CF.

The body CF percentage and the body abdominal fat pad weights increased between wk 20 and 35 in all hens. This indicates that overall, the energy intake was in excess of requirements and was stored into adipose reserve tissue. Fluctuations in-between weeks were not measured, so there could have been weeks of negative energy balance when body reserves were used. Nevertheless, in general it seemed that energy intake was sufficient to support growth and egg production during the early peak phase and that this was not influenced by rearing diet treatment.

### Allometric Growth Curves

Ontogenetic allometry can give insights into how various body components grow relative to each other (Gayon, 2015). By fitting multiphasic allometric relationships on an LN scale, different sections of linear growth are determined, separated by inflection points. When the slope is close to a value of 1, growth of both body components is isometric, that is, at a similar rate, whereas a slope smaller or larger than 1 indicates that one body component (x-axis) grows relatively slower or faster than the other (y-axis). These comparisons do not take into consideration the chronological age of the animals, but rather focus on the physiological and biological relevance for development. To facilitate practical interpretation of results, the corresponding chronological age of the phases and inflection points in this study were retrieved from the data and added to the discussion.

**Figure 1:**
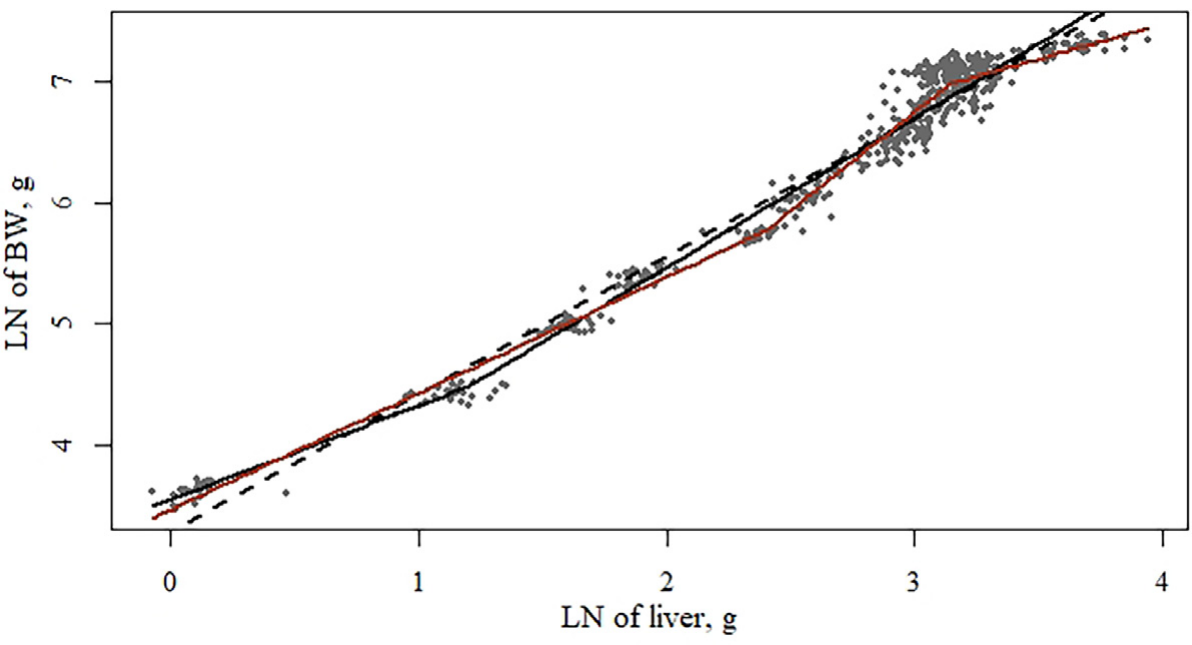
Growth allometry of LN body weight (g) and LN of liver weight (g) of pullets between 0 and 20 wk of age, fed 3 rearing diet densities. The dotted line (- - -) represents the fitted linear model, the straight black line (―) the fitted model with 1 inflection point and the straight red line (―) the fitted model with 2 inflection points. Each dot represents 1 replicate (pen).

***Liver.*** Liver function is crucial for long-term egg production (van Eck et al., 2023) and supporting its development during rearing might therefore be important. The current trial indicates that the initial phase of liver growth is isometric (value of 1) with BW, except when low density diets are fed, in which case the liver is growing at a slower rate than BW (Table 13; Figure 1). In the second phase, the liver is growing at a faster rate than the entire body, starting at approximately 4 wk of age. Also in this phase, the liver of pullets fed a low density diet grew at a relatively slower rate than the liver of pullets on the medium density diet treatment, with an in-between result of the high density diet fed pullets. The inflection point for the final phase of growth was significantly higher for the pullets fed the low density treatments. This corresponded to a rather large age difference, with the liver continuing to grow at a faster rate than the BW for the pullets fed the low density diets until on average 17 wk of age, but only 10 wk of age for the pullets fed the medium and high density diets. As a result, there was a tendency for a linearly heavier liver weight (in percentage of BW) for pullets fed the lower density diets at the end of the rearing phase continued toward the end of the trial in wk 35 (data not shown; liver weight of 1.93% for the low density diet vs. 1.81% for the high density diet).

The liver is an energy-supplying organ, so early development is important to sustain later body growth and egg production (Scheele, 1997). The pullets fed a low density diet showed both relative slower liver growth but also lower ADG. The slower growth rate of the liver tended to result in heaver relative liver weights at later ages, which could be beneficial to support egg production (Shivaprasad and Jaap, 1977). The low density rearing diets contained both lower crude fat and added soybean oil than the medium or high density diets. The difference in liver growth between the hens fed low and high density diets is not in line with expectations based on laying hen studies, in which lower soybean oil diets result in lower liver weights and fatty liver syndrome (Bragg et al., 1973). Liver development in response to diet might therefore differ between growing birds and egg producing hens. Liver composition was not measured in the current study but should be considered for future trials, as not only the liver weight but also crude fat level or fatty acid composition can have an influence on liver function and egg production (Bragg et al., 1973).

**Figure 2:**
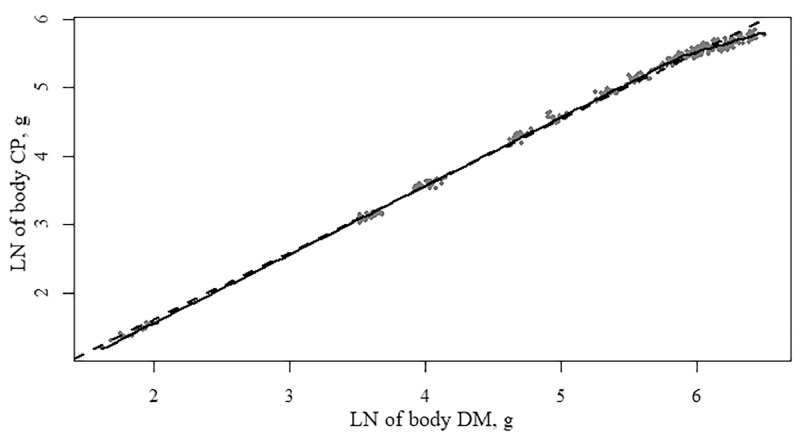
Growth allometry of LN body dry matter (g) and LN of body crude protein (g) of pullets between 0 and 20 wk of age, fed 3 rearing diet densities. The dotted line (- - -) represents the fitted linear model and the straight line (―) the fitted model with 1 inflection point. Each dot represents 1 replicate (pen).

***Body Composition.*** The results of this study showed that in pullets and young laying hens until 20 wk of age, the body CP initially grew isometric, with a slope of DM vs. CP close to 1 (Table 14; Figure 2). When the body LN of the DM reached a value of 5.9, the body CP started to grow slower than the body DM, with a slope of around 0.57. This corresponds to an age of approximately 12 to 14 wk. Body CP showed the best model fit with a 2 phase model with 1 inflection point, but was not influenced by dietary density. Interestingly, the data by Kwakkel et al. (1993) showed a very similar inflection point and slope in a 2 phase model, although they did not test for a 3 phase model. Also the comparison with body composition in the study by Alves et al. (2019) showed very similar body CP composition. This strongly indicates a stable body protein growth that was not influenced by genetic selection in the past 25 yr, breed or by dietary density, as found in the current study.

**Figure 3:**
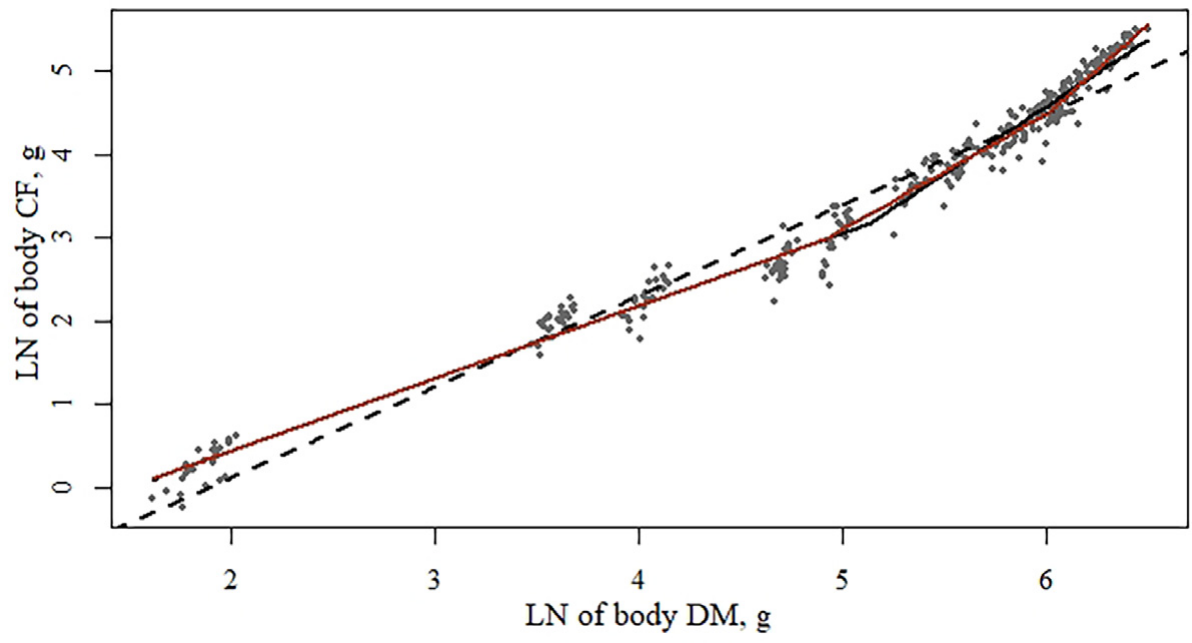
Growth allometry of LN body dry matter (g) and LN of body crude fat (g) of pullets between 0 and 20 wk of age, fed 3 rearing diet densities. The dotted line (- - -) represents the fitted linear model, the straight black line (―) the fitted model with 1 inflection point and the straight red line (―) the fitted model with 2 inflection points. Each dot represents 1 replicate (pen).

Body CF, on the other hand, showed complete allometric growth, starting with a slope of DM vs. CF smaller than 1, indicating that body fat was deposited at a relatively lower rate than the body growth (Table 15; Figure 3). The first inflection point was significantly lower for pullets fed a high density diet, with a significantly higher slope in the second phase as well. The first inflection point occurred at an age ranging between 2 wk of age (high density treatment) and 6 wk of age (low and medium density treatment). All 3 treatments showed relatively higher body fat deposition compared to body DM in the second phase, as indicated by a slope higher than 1; but for the pullets fed the high density diet, the second phase started earlier and the body fat deposition rate was higher. The second inflection point occurred at an age ranging between 8 wk of age (high density treatment) and 15 wk of age (low and medium density treatment). Also, a third phase was found, again with a lower inflection point for pullets fed higher density diets, but the third slope was no longer influenced by treatment diet. These results suggest that pullets fed higher density diets gained relatively faster body CF than pullets fed the low and medium density diets in a shorter, first phase of growth occurring before the hens were 6 wk of age. Comparing these results with the responses found by Kwakkel et al. (1993), the first inflection point in the current study was at a much lower LN of body DM (on average 4.9 vs. 5.5) and a lower LN body CF (3.0 vs. 4.2). Also the slopes show clear differences, with isometric growth of body CF found by Kwakkel et al. (1993), while this study showed allometric growth responses of body CF vs. DM already from an early age. In contrast to body CP, the growth pattern of body CF was not as stable over time and dietary changes determined the slope changes and inflection points.

**Figure 4:**
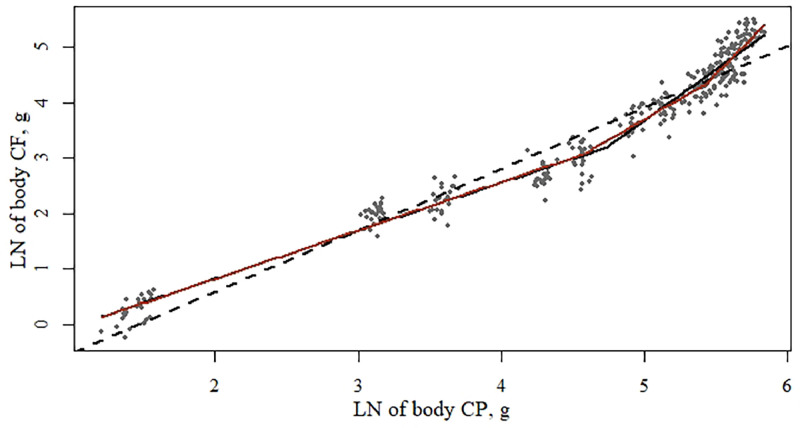
Growth allometry of LN body crude protein (g) and LN of body crude fat (g) of pullets between 0 and 20 wk of age, fed 3 rearing diet densities. The dotted line (- - -) represents the fitted linear model, the straight black line (―) the fitted model with 1 inflection point and the straight red line (―) the fitted model with 2 inflection points. Each dot represents 1 replicate (pen).

***Body CP Vs. CF.*** Comparing the growth of body CF vs. CP, a 2 inflection point segmented model showed the best fit, with a significant effect of rearing diet (Table 16; Figure 4). Until the first inflection point, the slope of body CF vs. body CP was smaller than 1 for the low and medium density diets, indicating that body CP grew at a relatively faster rate than body CF. For the pullets fed the high density diets, however, the slope was 1.09, indicating isometric growth of body CF and body CP. The inflection point was, on the other hand, significantly lower when pullets were fed a higher density diet, at a value of 3.5 corresponding to an age of 3 wk whereas the first phase ends at wk 6 for the pullets fed the low, and wk 5 for the pullets fed the medium density diets. Thus, a higher density diet increased the slope, that is, pullets had a similar fat deposition and protein deposition, and that next phase of growth started earlier. For pullets fed a low or medium density diet, the slope of the second and third phase was higher than the first phase, indicating that pullets started to grow body fat at a relatively higher rate than body protein. For pullets fed the high density diet, the slope of the second phase was, with a value of 0.13, almost flat. The slope of the third phase was not significantly different anymore. Even though the pullets fed the high density diets showed isometric growth of CP and CF in the first (shorter) phase, this was followed by a period of higher relative body CP vs. CF growth. This might indicate that pullets have some intrinsic compensatory body CP growth, or relative body CF reduction.

The difference in body CP and body CF growth was probably influenced by diet and genetics. Pullets fed the high density diets had a relatively higher energy and protein intake, but the energy to protein ratio was kept constant in all diets. The energy to protein ratio intake was therefore also similar for all treatments. Body protein seems to be predetermined by genetics and determining the growth and feed intake, since the breast percentage and body DM content were similar in all treatments and showed no changes when compared to 30 yr ago. A limitation in feed intake capacity might have limited the protein intake of hens fed the lower density diets, thereby limiting growth. The body CF percentage and the body abdominal fat pad weights were linearly or quadratically increased with diet density in most sampling points and at the end of the rearing phase. Excessive energy intake (as a consequence of hens eating on protein intake) was potentially converted into body fat. This makes body fat more flexible in development, that is, more influenced by diet density, than body protein.

### Study Limitations

To determine the body composition of the hens, 1 or 2 hens per experimental unit were dissected and no further growth or egg production data of these specific hens could be measured. This selection process probably added some variation, a challenge in these type of studies, that can be reduced by increasing the number of observations (Kwakkel et al., 1993) It is therefore assumed that the body composition of these dissected hens, at a specific point in time, are representative for all hens within that experimental unit and provide relevant information that can be linked to the future body weight and egg production data of the hens in the experimental unit.

### Practical Implications

The number of feeding phases for pullets have increased in breed recommendations and commercial diets over time. The current study shows, however, that a maximum of 2 phases of CP growth and 3 phases of CF growth compose pullet growth until 20 wk of age. Body DM and CP growth were isometric until approximately 12 to 14 wk of age, after which growth was more affected by body CF. If nutritionists aim to influence body CF development, this can already be done from an early age onward and especially after 5 to 6 wk of age, body CF grows at a relative faster deposition rate than body CP.

## CONCLUSIONS

Current results showed that feeding increasing diet densities during the rearing phase resulted in linear increased BW at the end of the rearing phase. Obtained BW differences were maintained during the peak production phase until wk 35, despite feeding a common lay diet to all treatments. Body weight was increased with a higher fat but not protein deposition. Egg production, egg weight, egg mass and FCR for egg mass until wk 35 were not affected by diet density during the rearing period. The breast percentage and body CP content were not influenced by dietary treatments, whereas the body CF and abdominal fat pads were linearly increased when pullets were fed higher density diets. Current results indicate that changes in diet density affect body CF deposition more than body CP deposition, from an early age onward.
